# Taxonomic and Feeding Trait-Based Analysis of Macroinvertebrates in the Antisana River Basin (Ecuadorian Andean Region)

**DOI:** 10.3390/biology12111386

**Published:** 2023-10-30

**Authors:** Santiago Cabrera-García, Peter L. M. Goethals, Koen Lock, Luis Domínguez-Granda, Marcos Villacís, Remigio Galárraga-Sánchez, Christine Van der heyden, Marie Anne Eurie Forio

**Affiliations:** 1 Laboratory of Environmental Toxicology and Aquatic Ecology, Department of Animal Sciences and Aquatic Ecology, Ghent University, Coupure Links 653, Block F, 9000 Ghent, Belgium; peter.goethals@ugent.be (P.L.M.G.); koen_lock@hotmail.com (K.L.); marie.forio@ugent.be (M.A.E.F.); 2 Facultad de Ingeniería en Ciencias Agropecuarias y Ambientales, Universidad Técnica del Norte, Av. 17 de Julio 5-21 y Gral. José María Córdova, Ibarra 100105, Ecuador; 3 Institute of Chemical and Environmental Sciences, Escuela Superior Politécnica del Litoral, Km. 30.5 Vía Perimetral, P.O. Box 09-01-5863, Guayaquil 090150, Ecuador; ldomingu@espol.edu.ec; 4 Department of Civil and Environmental Engineering, Escuela Politécnica Nacional, Avenida Ladrón de Guevara E11-253, Quito 170525, Ecuador; marcos.villacis@epn.edu.ec (M.V.); remigio.galarraga@epn.edu.ec (R.G.-S.); 5 Health and Water Technology Research Centre, Hogeschool Gent—University of Applied Science and Arts, Valentin Vaerwyckweg 1, 9000 Ghent, Belgium; christine.vanderheyden@hogent.be

**Keywords:** functional feeding groups, ecological conditions, BMWP-Col, Andean streams

## Abstract

**Simple Summary:**

High-altitude Andean streams are fragile ecosystems that require urgent actions such as bioassessment for proper environmental management. In the present study, we investigated the distribution and composition of the macroinvertebrate community in relation to the environmental variables in the Antisana river basin (Andean–Ecuadorian region). Macroinvertebrates were sampled at fifteen localities to predicate ecological condition which were expressed by the Biological Monitoring Working Party Colombia (BMWP-Col) index, the Andean Biotic Index (ABI) and the Andean–Amazon Biotic Index (AAMBI), and their main feeding traits as indicators of ecological functionality. Results show that dissolved oxygen saturation and total phosphorus, ammonia and conductivity contributed significantly to the composition of taxa and functional feeding groups (FFGs). Taxa and FFG diversity were most abundant at sites with the best ecological conditions. Shredders were, in general, dominant and especially abundant in the medium-high-quality sites. Predators were almost absent throughout the study, but they were collected from low-quality sites, showing greater tolerance to the presence of human disturbance. The BMWP-Col index seems to be the best fit for this ecosystem, showing a significant difference in FFG between the index classes compared to the other indices evaluated. The results of the present investigation may be regarded as a fundamental starting point and used in the future bioassessment works in other tropical Andean streams, especially where their resilience is threatened by poorly managed human activities.

**Abstract:**

High-elevation tropical streams are under increasing threat from human activities and climate change. Specifically, Ecuadorian Andean streams require priority actions such as bioassessment (e.g., biodiversity and functional ecology of macroinvertebrates) in order to generate adequate environmental management policies. Therefore, we investigated the distribution and composition of the macroinvertebrate taxa and their functional feeding groups in relation to the environmental variables in the Antisana river basin (Andean–Ecuadorian Region). We sampled macroinvertebrates from 15 locations to assess ecological conditions (ECs), expressed as the Biological Monitoring Working Party Colombia (BMWP-Col) classes, the Andean Biotic Index (ABI) and the Andean–Amazon Biotic Index (AAMBI). Results indicate that dissolved oxygen saturation, elevation, nutrient concentration and conductivity contributed significantly to the composition of the taxa and functional feeding groups (FFGs). Taxa diversity and FFGs were more abundant in the best EC sites. Shredders (SH) were, overall, dominant and abundant at sites with medium-high ECs. Scrapers constituted the second most prevalent assemblage, exerting dominance at moderate ecological conditions (high altitude and high oxygen saturation). Collector–gathers (CGs) are less sensitive to contamination than the previous two groups but were equally abundant at medium-high EC sites. Collector–filterers (CFs) and parasites (PAs) were less abundant, although the presence of the former was slightly related to better environmental conditions. Predators (PRs) were almost absent throughout the study, but they were collected from poor EC sites. CGs, PAs and PRs showed more tolerance to the presence of human disturbances (e.g., hydraulic constructions or slope erosion). The BMWP-Col index seems to be the best fit for this ecosystem, showing a significant difference in FFG between the index classes, compared to the other indices evaluated. The results of this investigation may be regarded as a fundamental starting point and used in future bioassessment work in other similar ecosystems, particularly high-altitude tropical Ecuadorian streams.

## 1. Introduction

Tropical high-altitude streams are ecosystems characterised by climatic extremes that generally have low temperatures, conductivity and nutrient concentrations, but high turbidity due to the presence of suspended solids [1]. The temperature of these streams can be as cold as their counterparts at higher latitudes, although seasonality is much higher in temperate zones [2]. Because of the lack of seasonality, the melting and ablation of equatorial glaciers can occur throughout the year, and the main discharges from these streams occur daily due to nocturnal freezing and diurnal melting [3,4]. A particularly interesting consequence of such extreme altitude is that the oxygen concentration remains constant or even declines due to the decrease in atmospheric pressure, which counteracts the effect of the decrease in water temperature and solubility [2,3].

The terms Andean, mountain, highland or upland streams generally refer to streams with characteristics such as steep slopes, rapid and turbulent flows and coarse substrates [2,5,6]. Some authors indicate that streams above 3000 m.a.s.l. can generally be referred to as “high altitude” [2,4,7,8]. In the northeastern region of the Andes, the relatively humid and species-rich vegetation is known locally as “*paramo*” [6]. The relationship between altitude and precipitation is often determined by local topography, degree of continentality, latitude and wind patterns [4,6,8]. As a general rule, precipitation in high-altitude areas is lower than at lower elevations and may fall as drizzle, sleet, hail or snow, but usually not as torrential rain, which is typical for tropical lowlands [2].

High-altitude tropical environments can be severely affected by anthropogenic activities due to the susceptibility of their biota to oxygen depletion [2]. Although human population density tends to decrease at higher altitudes, human activities may extend to higher altitudes, due to the relatively benign climate in this region [9,10]. Andean-tropical streams are a vital source of water for human activities in the downstream stream section [11]; however, these streams and drainages can be polluted by a range of anthropogenic inputs, such as organic and microbiological pollution, as well as nutrients, sediments and pesticides [2,10]. Some impacts (especially from human beings) are caused or enhanced by global climate change [12]. Andean glaciers, like those in the rest of the world, are retreating with profound effects on the water regime [1,13]. Also, water abstraction reduces stream discharge (or causes its complete disappearance) [9]. All these impacts and others (e.g., erosion, construction or fragmentation) affect physical habitats, water quality and, consequently, ecological function and community structure [1,2,10].

Information on tropical high-altitude environments is scarce and scattered, including biogeochemical cycles and climatic and physico-chemical conditions, but mainly stream ecology [2,3,14,15]. Most research postulates have been generated based on temperate–arctic environments, and the similarities and differences with Andean-tropical streams [12,15,16,17]. Since high-altitude streams present harsh conditions, they act as environmental filters excluding many aquatic species in these habitats [1]. Some insights into the distribution of natural aquatic community composition indicate that the density and richness of benthic macroinvertebrate taxa normally decrease with increasing glacier cover [16], and the reduction in species richness along the altitudinal gradient (from low to high) is a clear trend in many species groups [18]. Patterns have been explained in terms of reduced ecosystem productivity, temperature and available oxygen and/or physiological stress imposed by a harsh climate [2,19,20,21]. Despite how little is known about the ecology in tropical high mountain streams, they appear to follow those known from higher latitudes [22,23]. Changes that occur at the level of climatic, physical or environmental factors would be expected to occur in biological communities (i.e., macroinvertebrates in the present study), but even if there are changes in the taxonomic composition of the community, this does not necessarily reveal changes in ecological function [1,24]. The distribution of biological and functional traits, such as the feeding habits of macroinvertebrates, have shown patterns due to the climatic characteristics of high Andean tropical environments. For example, the degree of omnivory seems to increase with glacial influence [17].

Scientists have implemented indices of tolerance or sensitivity to environmental pollution in rivers using macroinvertebrates as bioindicators, which can be utilized for a rapid and efficient evaluation of the river’s condition [25]. The utilization of these indices is based on three fundamental assumptions. Firstly, a healthy river is characterized by a diverse and heterogeneous fauna, comprising numerous species. Conversely, a polluted river will exhibit reduced diversity and the dominance of a few species that are tolerant to adverse conditions. Secondly, different taxa, such as families, possess varying levels of environmental tolerance. Consequently, categorizing invertebrates based on their tolerance or sensitivity levels aids in determining the overall health of the river. Lastly, the effectiveness of these indices is contingent upon accurate comparisons and the presence of a reference or control ecosystem. The reference or control sites should ideally be devoid of significant anthropogenic pressures, ensuring that the diversity of organisms closely resembles the natural evolution of the system in its pristine state [26,27]. In the Andean mountain ranges, a multitude of studies have utilized macroinvertebrates as biological indicators [2,5,15,28]. Modified versions of the Biological Monitoring Working Party (BMWP) index have been applied in several of these studies. Colombia and Argentina have developed their own preliminary adaptations of the index, which have been utilized with minimal modifications in other South American countries [29,30]. Other researchers have proposed biological indices that they deem more accurate in reflecting the conditions present in high-altitude tropical ecosystems. The Andean Biotic Index (ABI) and the Andean–Amazon Biotic Index (AAMBI) are examples of such indices, which aim to better represent the ecological conditions of tropical Andean and Amazonian streams. Furthermore, these indices strive to incorporate the appropriate pollution ranges for each macroinvertebrate family, utilizing more precise values as improved tools for the Andean and Amazonian region [25,26].

The complexity of macroinvertebrate communities is better studied from a holistic approach, integrating the structural and functional perspective [31]. On the one hand, diversity provides an insight into habitats by species and constitutes a good perspective when analysing biodiversity in stream systems [32] and on the other hand, if the aim is to determine the role of species in the ecosystem, functional feeding groups (FFGs) may be appropriate, as they simplify the benthic community into trophic guilds [33,34]. The distribution of FFGs in running waters is assumed to reflect the process-level attributes of aquatic ecosystems [22]. Previous studies have demonstrated the predictions of the River Habitat Templet concept regarding the macroinvertebrate species’ response to fluctuating environmental conditions in streams. Specifically, the concept indicates that appropriate biological and ecological responses depend on the constraints on their physiological, morphological and behavioural characteristics [14,15,35,36,37,38]. For some years now, feeding habits have been used in biomonitoring processes such as the “Benthic Index of Biotic Integrity” [39] or the “Rapid Bioassessment Protocols” [40]. However, the challenges in tropical high-altitude streams involve the lack of specific information about the FFGs of aquatic invertebrates. As Covich [41] and Tomanova et al. [34] pointed out, the highly flexible life histories and mobility that seem to characterise tropical stream taxa may influence their flexibility in obtaining food and this may produce a significant difference in FFG classification that may not feed as their temperate-dwelling cogenerates, so despite having already worked out some classifications for the region (i.e., [42,43,44,45,46,47,48,49]), there may still be some bias.

Thus, the present study aims to contribute to the knowledge of the ecology and dynamics of macroinvertebrate Andean species. It is postulated that locations exhibiting a minimal geographical separation are characterized by comparable macroinvertebrate assemblages, both in terms of FFG and taxonomic classification. To that end, we set the following objectives: (i) identify potential physico-chemical and hydro-morphological influential variables, and assess the ecological condition, expressed as the BMWP-Col, ABI and AAMBI; (ii) describe the similarities among sampling sites based on the family composition, abundance and richness and the functional feeding group distribution; and (iii) determine the differences in macroinvertebrate families and FFG composition among BMWP-Col, ABI and AAMBI classes. The aim of the present study is to contribute to the scientific understanding of macroinvertebrate communities and their feeding habits in areas considered pristine and protected ecosystems that provide some ecosystem services to human beings (i.e., drinking water supply, water recharge, ecotourism). Finally, all this knowledge would allow the adoption of the most cost-effective and suitable water management measures, including climate change adaptations and decisions [50].

## 2. Materials and Methods

### 2.1. Study Area

**Figure 1:**
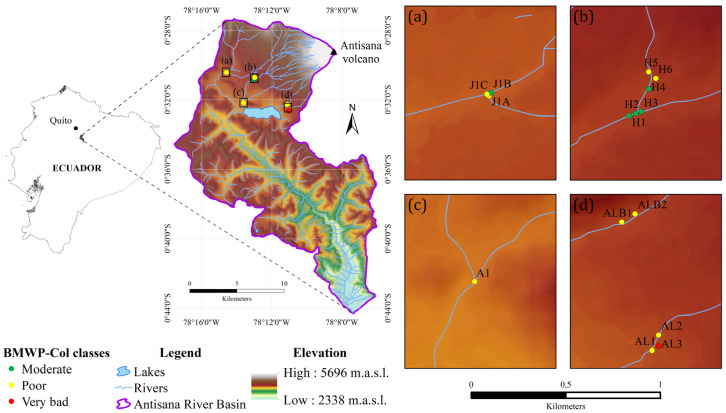
Map of the study area with sampling sites and its BMWP-Col classes. Letters (**a**–**d**) represent a zoom of the sampling sites within the Antisana River Basin. The sampling site codes used were: A1 (Antisana), AL1-AL2-AL3 (Moyas stream), ALB1-ALB2 (Alambrada stream), H1-H2-H3-H4-H5-H6 (Humboldt stream) and J1A-J1B-J1C (Antisana stream).

We executed a sampling campaign in the Antisana river basin (330.42 km^2^) during the wet season of July 2014 [51]. This river basin is located on the southwest slopes of the ice-capped Antisana volcano in the Ecuadorian Andes (Figure 1) and is a National Park [51,52]. Due to its geographical location, the Antisana National Park is rich in water resources. Its wetlands, the lagoons of its *páramos* and the formation and water retention lagoons such as La Mica, Papallacta and its montane forests, supply drinking water to the city of Quito and a large part of the metropolitan area [52]. Perpetual snow and tropical rainforest are combined in this Antisana National Park [1]. Our sampling sites specifically are located near the top of the volcano, due to the special characteristics of this altitude on ecological and abiotic conditions. The climate is distinguished by a consistent and substantial amount of precipitation throughout the entirety of the year but with low intensity. The annual precipitation during the hydrological year ranges from 800 to 3000 mm [51]. During almost all months of the year, there is cloudiness except in the driest months of November, December and January, since this is the time of greatest solar radiation [51]. The temperature in the reserve ranges from 3 to 17 °C, with temperatures rarely exceeding 20 °C above 3000 m.a.s.l. [53]. Data obtained from a weather station located at La Mica Lake for the 2000–2010 period, indicated that the daily temperature had high variations ranging from 5.5 to 18 °C, which prevents frost, snow and hail from staying on the ground for periods longer than 2 or 3 days and, at the same time, prevents the water in the streams and the lake from freezing [51]. The Antisana River Basin (ARB) drains the main river through 26 streams, which has an extension of 13.72 km and discharged an annual average of 795.69 L/s during the 2008–2017 period [51,52].

Fifteen sites were selected. The sites were in the following streams: Antisana (A and J1), Moyas (AL), Alambrada (ALB) and Humboldt stream (H) (Figure 1). The sites were at similar altitudes near the highland in the Andes (Antisana volcano) from 3900+ to 4000+ m.a.s.l. Sampling sites were chosen primarily on the basis of their level of accessibility and junctions of several small streams. In the upper part of the basin, almost all the water is collected in the La Mica reservoir for sending to Quito via a pipeline [51]. Therefore, it was interesting to test influents from different locations. We assumed that the water quality and ecological conditions would be excellent given the pristine conditions of these ecosystems and for being part of an ecological reserve.

### 2.2. Physico-Chemical Analysis

**Figure A1:**
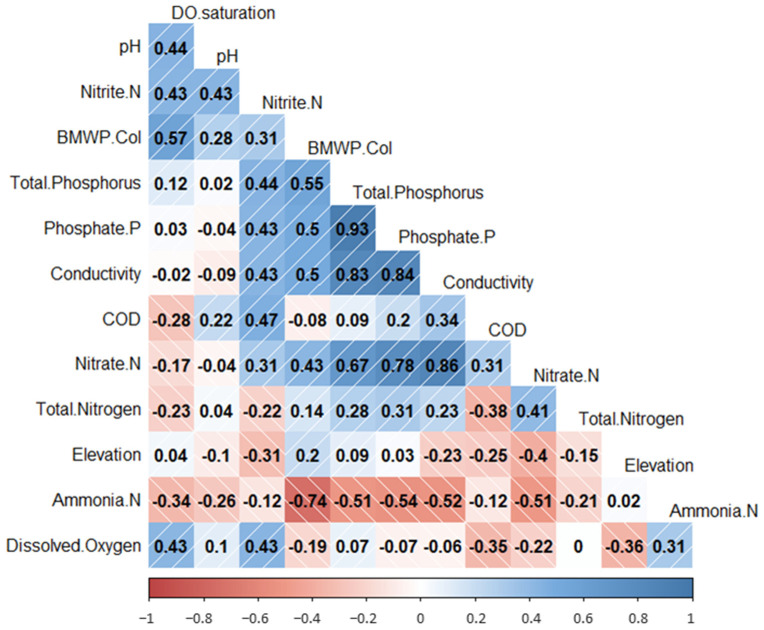
Spearman’s rank correlation diagram of 13 environmental variables of the Antisana river basin. Variables are organized according to their degree of correlation. The background lines and colour intensity indicate whether the correlation is directly or inversely proportional and the degree of association, respectively.

**Figure A2:**
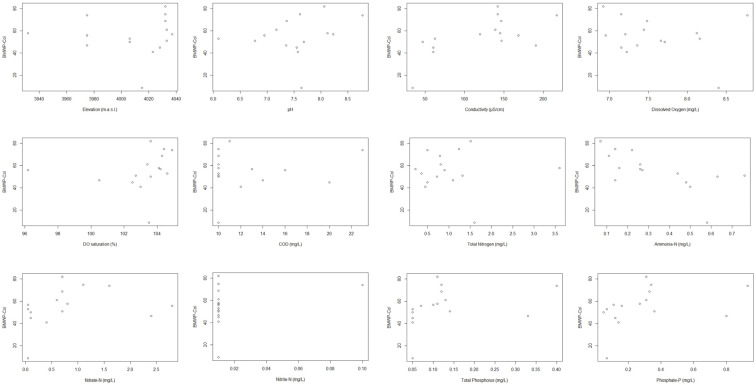
Plots of physico-chemical variables in relation to BMWP-Col for sampling sites in the Antisana river.

In situ measurements of dissolved oxygen (DO), dissolved oxygen saturation, conductivity and pH were conducted using a field probe (Three-Multi 3430 IDS, WTW GmbH, Singapore). We also measured water temperature, but since measurement times varied considerably throughout the day, the water temperature reflected the variation during the day rather than the differences between study sites; thus, this parameter was removed. The measurements were performed indirectly in a bucket previously filled with water from the stream. Water samples were obtained from the midchannel in order to ascertain the levels of chemical oxygen demand (COD) and nutrient content. The water samples were placed in plastic bottles for nutrient analysis, and they were stored in a cooling box and then placed in the freezer upon arrival in the housing facility. Then, they were transported frozen to the lab and were analysed immediately after arrival. The COD and nutrient analysis were carried out in the ‘Centro de Investigación y Control Ambiental’ in the Escuela Politécnica Nacional (EPN) in Quito following the same procedure as described in Cabrera et al. [38]. To measure coordinates and elevation, a Garmin Etrex GPS (Global Positioning System) equipment (Garmin Legend; Garmin Ltd., Olathe, KS, USA) was used. Flow velocity at each site was measured in the midchannel at least three times at different points, equidistant through the stream reach, using a handheld OTT probe (C2-model; OTT HydroMet, Sterling, VA, USA). Additionally, the width and depth, as hydro-morphological variables of the streams, were measured and assessed 3 times at different transects. Wetted or bank width was not measured. In order to examine the association among the main variables, as well as their relationship with the BMWP-Col index, a Spearman correlation analysis was conducted and visually represented (Appendix A Figure A1 and Figure A2).

### 2.3. Macroinvertebrate Data Collection

The macroinvertebrates were captured from each sampling site after establishing the physico-chemical and hydro-morphological variables. The standard hand net is composed of a metallic framework that securely holds a conically shaped net (mesh size 500 µm) was used to collect the aquatic invertebrates. Macroinvertebrates were collected via the kick-net sampling procedure for 5 min by one person trying to cover the different microhabitats present at each sampling site. The sampling effort is proportionally distributed over all accessible aquatic habitats [54]. The macroinvertebrate sample was subjected to sieving using a mesh size of 500 µm at the identical sampling sites. Subsequently, the sorted macroinvertebrates were placed in individual white trays. To achieve a final concentration of 70%, the macroinvertebrates from each site were transferred into separate 10 mL tubes containing 96% ethanol. After sorting, all invertebrates were identified and their abundances calculated under a stereomicroscope [54]. These communities were identified using the identification guides developed by Domínguez and Fernández [55]. Identification was conducted at the family level for two primary reasons. Firstly, the absence of comprehensive keys hinders identification to lower taxonomic levels. Secondly, prior research has demonstrated that utilizing biotic indices founded on the family level provides adequate information for evaluating ecological condition [56,57,58].

### 2.4. Calculation of Ecological Condition Indices for River Assessment

The ecological conditions of each sampling location were evaluated using the Biological Monitoring Working Party (BMWP) index, which was adapted for Colombia and referred to as BMWP-Col [30]. This index was employed due to the absence of a macroinvertebrate-based ecological condition index specific to Ecuador. The BMWP-Col was based on the work of Álvarez [29]. Previous studies, including those conducted by Dominguez-Granda et al. [56], Damanik-Ambarita et al. [42] and Cabrera et al. [38], also utilized the BMWP-Col to assess the ecological condition of the Chaguana, Guayas, and Aguarico/Coca river basins in Ecuador, respectively. This is because this index is considered appropriate for Ecuador given the relatively similar ecological, orographic, geographic and climatological conditions it shares with Colombia [59]. The BMWP-Col calculation was formulated utilizing the composition of macroinvertebrate communities, specifically their presence or absence. Each taxonomic group of invertebrates is associated with a designated tolerance score, ranging from 1 to 10. Higher scores indicate the presence of sensitive taxa, while lower scores indicate the presence of tolerant taxa. The ecological quality of a site can be assessed based on the BMWP-Col score, with values of ≥100, 61–100, 36–60, 16–35 and 0–15 representing good, moderate, poor, bad and very bad ecological quality, respectively [29]. Since the Antisana sites are so high and since they are qualified as Andean equatorial streams, we also calculated the ABI (Andean Biotic Index) and AAMBI (Andean–Amazon Biotic Index) indices, which have approaches closely related to those of the BMWP-Col; i.e., the form of calculation based on the aquatic invertebrate tolerance is ranked from 1 to 10, although with slight differences in the macroinvertebrate families and the classes and proposed quality values used. An ABI score of >96, 59–96, 35–58 and <35 represents very good, good, regular and bad, respectively. Meanwhile, an AAMBI score of >121, 90–120, 50–89, 36–49 and <35 corresponds to excellent, very good, good, regular and bad ecological conditions, respectively [25,26].

### 2.5. Feeding Habit Trait Allocation

Information on the main functional traits associated with macroinvertebrate communities was compiled from different databases [42,43,44,45,46,47,48,49]. Given that every macroinvertebrate taxon was meticulously identified up to the family level, which typically represents the highest level of classification in the trait database, we employed the feeding habit traits of the prevailing and/or influential species encountered, as determined by the taxonomic specialist. We only focussed on feeding habit, as this trait (i) is often analysed, probably due to the relationship between ecosystems functions and feeding strategies of aquatic invertebrates [60] and (ii) has been considered an essential factor in the classification and composition of macroinvertebrate communities [61]. Each taxonomic group was allocated a distinct functional feeding group (FFG) based on the previously referenced databases. Subsequently, the abundance of each taxonomic group was designated as the abundance of the FFG linked to that particular group.

### 2.6. Data Analysis

#### 2.6.1. Environmental Variables and Functional Feeding Group Assemblages Compared between Sites Based on Ecological Condition Indices

To estimate the degree of association graphically and statistically between the study sites, the hydro-morphological and physico-chemical variables, the BMWP-Col, ABI and AAMBI indices and the FFG, a non-metric multidimensional scaling (NMDS) analysis was executed. NMDS analysis was used to show the changes in FFG abundance and hydro-morphological and physico-chemical variables in relation to biological indices. The NMDS is a robust ordination technique that produces an ordination based on similarity or dissimilarity among sites. The NMDS attempts to represent the pairwise dissimilarity between the sites in a reduced number of dimensions, as closely as possible using Bray–Curtis dissimilarity [62]. The sampling sites that are more similar to one another in terms of biological assemblages are ordinated together. Permutational multivariate analysis of variance (PERMANOVA) was used to compare the differences in environmental variables and FFG composition between the different indices classes [63]. A post hoc test on significant PERMANOVA (FDR-adjusted *p* ≤ 0.05) was also performed. NMDS, PERMANOVA and post hoc pairwise comparisons (function Pairwise Adonis) analyses were executed in R-Studio via vegan and Pairwise Adonis packages [63,64].

#### 2.6.2. Macroinvertebrate Community and Functional Feeding Groups

To investigate the relations of macroinvertebrates and functional feeding group (FFG) composition with BMWP-Col, the macroinvertebrate community and functional feeding groups composition (presence/absence) were graphically ordered according to an increase in BMWP-Col scores in the function of BMWP-Col classes (from worse to better ecological conditions) at sampling sites in the Antisana River basin. Moreover, the absolute and relative abundance of each functional feeding group (FFG) and its corresponding richness were computed at every sampling location. Subsequently, a stacked bar chart was generated for each ranked sampling site, depicting the absolute and relative abundance of each FFG and its relative richness. Ranking was developed from low to high ecological conditions, expressed as BMWP-Col.

#### 2.6.3. Other Ecological Quality and Trait Indices

To determine the relations of Rao index and other ecological indices, the Rao’s quadratic entropy was also calculated, based on the abundance of each taxon and the description of each taxon by their FFG [65]. Rao index calculation was executed in R-Studio using the syncsa package [65]. Subsequently, a matrix with each study site and its biological indices (BMWP-Col, AAMBI and ABI) and Rao index was developed to observe the existing qualitative and quantitative differences. Finally, to obtain the statistical and graphical association between the indices, a linear correlation was performed.

## 3. Results

### 3.1. Physico-Chemical Analysis

The values of all measured physico-chemical variables are presented in Table 1. The lowest conductivity was observed at the Moyas site-AL3 (34 µS·cm^−1^), while the highest was at a small tributary of Antisana stream-J1B (217 µS·cm^−1^). Conductivity also had a positive correlation (r = 0.83, *p* = 0.0001) with both total phosphorus, ${\mathrm{NO}}_{3}^{-}$ (r = 0.86, *p* ≤ 0.0001) and ${\mathrm{PO}}_{4}^{3-}$ (r = 0.84, *p* ≤ 0.0001). The pH ranged from 6.1 to 8.8. The streams with the slowest flow velocity, namely the Humboldt5-H5 and the Antisana-J1C, exhibited measurements of 0.24 m·s^−1^ and 0.28 m·s^−1^, respectively. These locations also demonstrated the lowest levels of dissolved oxygen (DO) saturation and DO concentration. At Humboldt1-H1 and H4, the highest flow velocity was found (1.37 and 1.16 m·s^−1^, respectively). DO ranged between 6.9 and 8.8 mg·L^−1^. The lowest oxygen concentration was observed at the Humboldt2-H2 site. In this basin, total phosphorus ranged from 0.05 to 0.40 mg·L^−1^, with the lowest value observed at some locations (AL and ALB sites). At J1B (northwest ARB), the highest value of TP, ${\mathrm{PO}}_{4}^{3-}$, ${NO}_{2}^{-}$, COD, pH, conductivity, DO and DO saturation were observed. There was a significant positive association (*p* ≤ 0.0001) between TP and phosphate (r = 0.93). NH_3_ ranged between 0.07 and 0.76 mg·L^−1^. The highest nitrate concentration (2.80 mg·L^−1^) was observed at J1C (slowest flow velocity site), while the lowest was in two Moyas sites, namely AL1 and AL3, and Humboldt5-H5 (0.05 mg·L^−1^). Nitrate also had a significant positive correlation (r = 0.78, *p* = 0.0006) with phosphate. Figure A1 and Figure A2 present the Spearman correlation coefficients between variables.

### 3.2. Ecological Condition Indices for River Assessment

**Figure 2:**
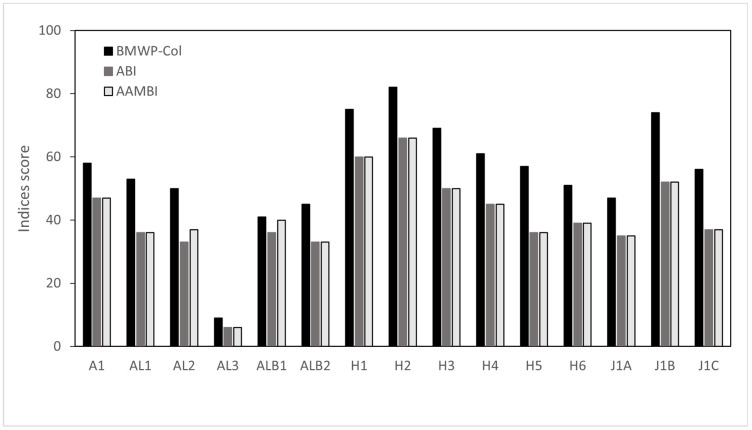
Scores of the ecological condition indices Biological Monitoring Working Party (BMWP-Col), Andean Biotic Index (ABI) and Andean–Amazon Biotic Index (AAMBI) of the different sampling sites in the Antisana river basin.

In total, 1745 macroinvertebrates belonging to 20 different families were sorted and identified. The highest abundance and richness were observed at Humboldt and Antisana stream sites (north ARB), each containing more than 100 individuals (except H5 site) and belonging to 8 and 13 different families, respectively. Hyallelidae, Baetidae and Dugesiidae were the most repeatedly encountered taxa (14, 15 and 13 sites, respectively). Similarly, Hyallelidae and Baetidae were the most abundant families, followed by Leptoceridae (in total 494, 458 and 227 individuals, respectively). Appendix A Table A1 displays the inventory of identified taxa, along with their respective abundance within the basin and the count of sampling sites where they were observed. The ecological conditions of the 15 sampling sites were assessed using the BMWP-Col index, with values ranging from 9 to 82 (Figure 1 and Table 2). The sites with a high abundance and richness of species exhibited a moderate ecological condition, which was deemed the most favourable. Despite the application of Spearman’s rank correlation coefficients to examine the relationship between physico-chemical parameters and the BMWP-Col, the coefficients were generally low (Figure A1), we found a significant positive correlation (*p* = 0.03) with total phosphorous (r = 0.55) and a negative association with ammonia (r = −0.74, *p* = 0.001). It was also observed that BMWP-Col had high values at sites with a slightly high altitude and DO saturation. The index had low values at the Moyas 3 (AL3) and La Alambrada (ALB1 and ALB2) sites, all located in south ARB (Figure 1). The ABI and AAMBI indices both ranged between 6 and 66, with the maximum and minimum coinciding in the same sites as the BMWP-Col. Most of the sites (11 out of 15) had a fair quality. Unlike the BMWP-col index, five sites reached the best ecological quality; only sites H1 and H2 had a good quality (60 and 66, respectively), according to the ABI index. Meanwhile, in the AAMBI index, four sites had the best quality (H3, J1B, H1 and H2). H1 and H2 were the only sites that obtained the best ecological quality in all three indices. The AAMBI index had the most sites with the worst quality, namely AL3 (6), ALB2 (33) and J1A (35), categorized as poor. AL3 was the only site with the worst ecological quality in all three indices (Figure 2). The RAO index ranged from 0.14 to 0.48, with the lowest values coinciding with the sites with the worst ecological quality, specifically site AL3. The sites with the best RAO values A1 (0.48), H6 (0.47) and H5 (0.45) did not coincide with the sites with the best ecological quality in the other three indices (Table A1).

**Figure 3:**
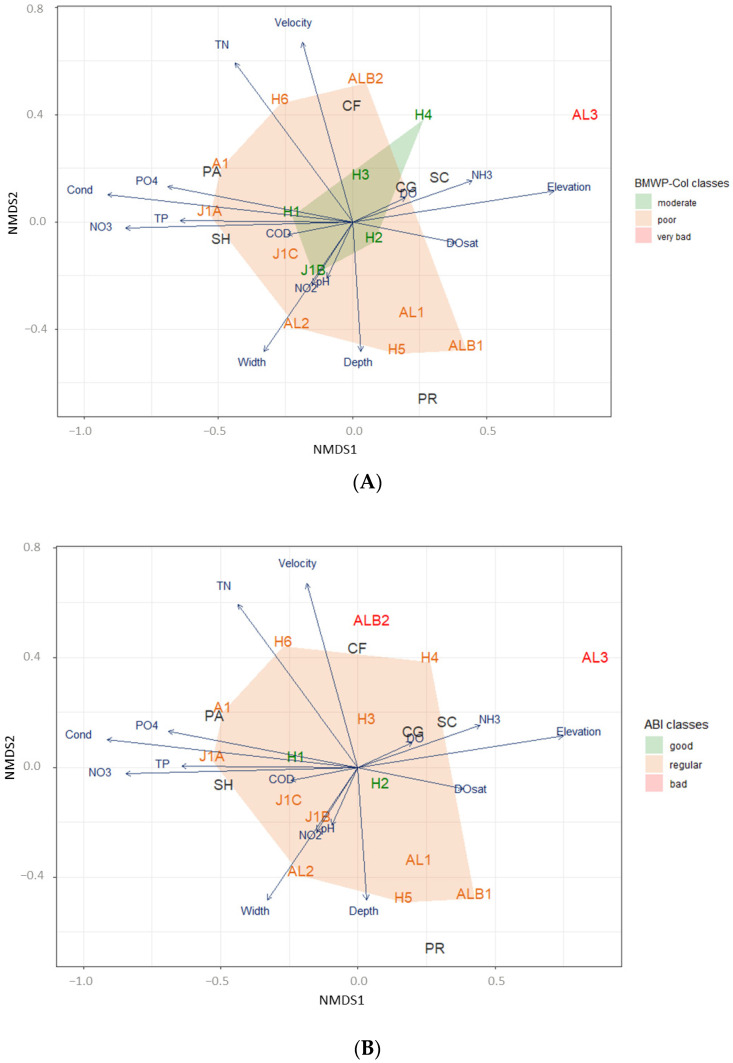

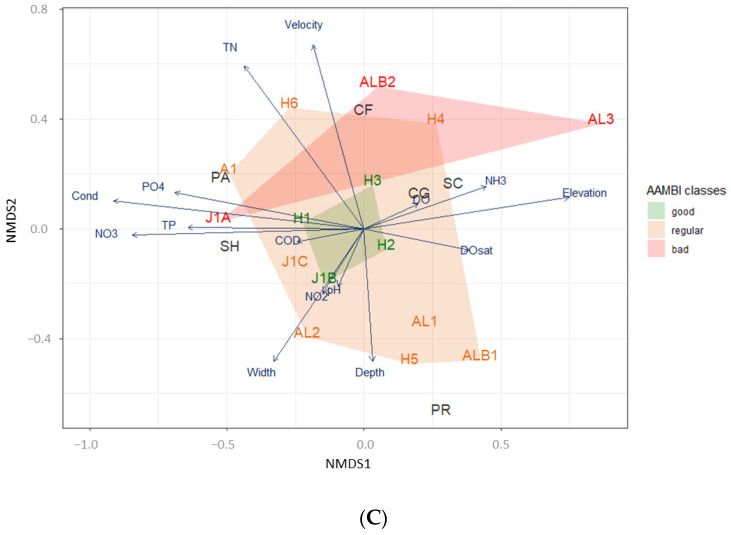
Non-metric Multidimensional Scaling (NMDS) diagram depicting the freshwater rivers located within the Antisana River basin (ARB), which were sampled in July 2014. The diagram illustrates the associations between the various sites based on their respective classes within three indices: (**A**) Biological Monitoring Working Party-Col (BMWP-Col) index, (**B**) Andean Biotic Index (ABI) and (**C**) Andean–Amazon Biotic Index (AAMBI). The diagram utilizes different colours to represent the ecological condition of the sites, thereby facilitating the visual interpretation of the associations. The main physico-chemical parameters are indicated in blue letters (see Table 1), where TN = Total Nitrogen; PO_4_ = Phosphate; NO_3_ = Nitrate; Cond = Conductivity; TP = Total Phosphorus; Velocity = flow velocity; DO = Dissolved Oxygen; NH_3_ = Ammonia; DOsat = Dissolved Oxygen saturation; COD = Chemical Oxygen Demand; and NO_2_ = Nitrite. FFGs are represented in black letters, where PA = parasites; CF = collector–filterers; SH = shredders; SC = scrapers; PR = predators; and CG = collector–gatherers.

Figure 3A shows that the worst ecological condition, expressed as the BMWP-Col score, is related to high ammonia and elevation (site AL3). It also appears that the wider, deeper and nitrogen-concentrated a stream is (>2.5 m), the worse its ecological condition, as observed at the sites AL2, H6 and AL1 (poor ecological condition). The locations with the highest index values are related to high values of DO saturation. Examples of this were the Humboldt locations (H2 and H3). It is also observed that the NMDS analysis seems to indicate a very close inverse relationship between flow velocity and depth. Sites with a high depth (i.e., ALB1, J1C or H5) had a low flow velocity and, therefore, a relatively low oxygen concentration and a poor ecological condition. Site AL3 is really a concrete channel, without vegetation, where the macroinvertebrates settle. It was observed to be totally isolated from the rest (Figure 3A) due to a very poor ecological condition, as only two taxa and 14 individuals were found there (Table 2).

The sites with better ABI scores were also related to oxygen concentration and organic matter, while the sites with lower ecological quality were also related to ammonia concentration and flow velocity, as shown in Figure 3B of the NMDS graph. With respect to the AAMBI index, the results showed that the study sites were related to the same environmental variables as the ABI index. And in the case of site J1A, which had a poor quality for the AAMBI index, it was also related to nutrient concentration and high conductivity (Figure 3C). The PERMANOVA analysis (Table 3) showed that there is no significant difference (*p* < 0.05) between the classes of all the ecological condition indices according to the environmental parameters indicated in Figure 3.

### 3.3. Ecological Conditions and Functional Feeding Groups

The NMDS analysis showed that the sites with better BMWP-Col ecological conditions (H2, H3 and H4) were characterized by a high abundance of scrapers, collector–filterers and collector–gatherers (Figure 3A). Collector–filterers were only represented by one taxon (Simuliidae), while scrapers and collector–gatherers had four and six taxa, respectively. In general, when only presence/absence was taken into account, there seemed to be some clear patterns with respect to the taxa found in the ARB (Table 2). The scraper, shredder and collector–gatherer species found have a high sensitivity score on the BMWP-Col biological index, and none of the species with these feeding habits have a sensitivity score less than 6, while the collector–gatherer and predator species found have a low sensitivity on this index, although two species (Leptoceridae and Hydrobioscidae) score high in the index. The collector–filterers, shredders and scrapers seemed to be more abundant in locations with a moderate ecological condition, especially the families Simuliidae, Limnephilidae, Hydroptilidae and Gripopterygidae, which are those with the highest sensitivity score in the BMWP-Col index (7, 8, 8 and 10, respectively). There were only three occurrences of these families in the very poor ecological condition sites. Collector–gatherers and predators showed a relatively more dispersed presence among the sites, although they were more distributed in areas with poor and very bad ecological conditions. Upon examining the distribution of macroinvertebrate species’ presence or absence, as well as their functional feeding groups (FFGs), in relation to the ecological condition classes of the BMWP-Col index at the sampling sites, it became apparent that greater trait diversity was associated with higher-quality conditions. Specifically, scrapers and shredders were found to be the dominant FFGs (Table 2).

**Figure 4:**
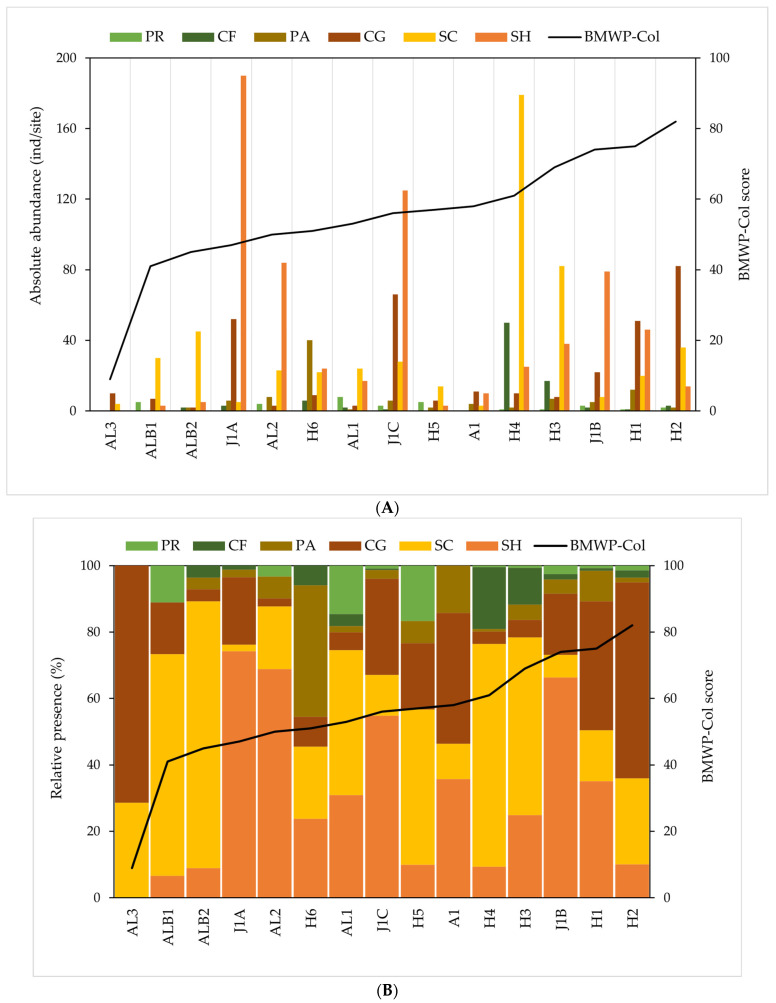

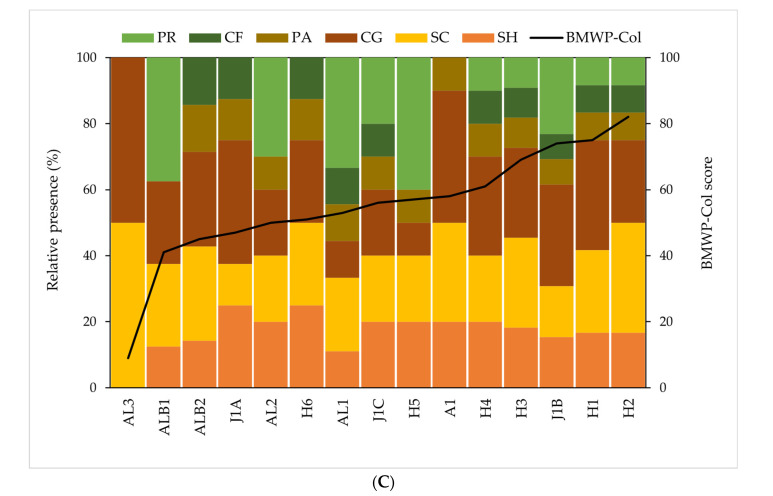
Functional feeding group composition per studied sites. Locations are ordered based on an increasing BMWP-Col score from bad to better ecological conditions. (**A**) Absolute abundance of macroinvertebrates with different traits. (**B**) Relative presence of macroinvertebrates with different traits. (**C**) Relative presence of macroinvertebrates trait richness per site. CF, SH, SC, PA, PR and CG refer to the collector–filterers, shredders, scrapers, parasites, predators and collector–gatherers, respectively.

NMDS analysis showed that DO saturation is positively correlated to high-BMWP-Col locations (Figure 3A and Figure A1). Figure 4 indicates that there was a higher richness and diversity of FFGs at sites with higher biological indices. The sites with the best BMWP-Col scores, namely H2 (BMWP-Col score: 82), H1 (75), J1B (74), H3 (69) and H4 (61), had a high taxa richness (12, 12, 13, 11 and 10, respectively). As indicated in Figure 4, the most abundant FFGs in these locations were scrapers, shredders and collector–gatherers. Some important characteristics could be distinguished among the locations with the best ecological conditions (Figure 4B—right), although they share similar characteristics such as a low presence of predators and parasites and high diversity. These important characteristics can be summarised in three groups: (i) H2, with the highest biological index score, a community dominated by collector–gatherers and a richness of 12 families containing in total 139 individuals (Figure 4A); (ii) H1 and J1B, with a joint dominance between shredders and collector–gatherers, an average abundance of 125 individuals (Figure 4A) and a richness of 13 families (Figure 4C); and finally (iii) H3 and H4, with a clear dominance of scrapers and the highest abundance of collector–gatherers of all study sites (67 out of 87 individuals) and an average abundance and richness of 210 individuals and 13 families, respectively (Figure 3).

When contributing to higher values of the ecological condition index BMWP-Col, it seems that richness is a more important factor than the density of organisms. Within the poor ecological condition, this situation was observed in sites A1 (BMWP-Col score: 58) and H5 (57), which despite having an abundance of only 28 and 30 individuals, respectively, were composed of an average of 10 families and reached better scores than sites H6 (56) and J1A (47), which had an abundance of individuals of 101 and 256, respectively, with an average of eight families (Figure 4). On the other hand, although the diversity of FFGs was similar to the sites with better ecological conditions, the families that composed poor-ecological-condition sites had a lower sensitivity score in the index (i.e., collector—gatherers—*Lumbricidae*—1, or predators—*Muscidae*—4) (Table 2). Shredders clearly dominated sites with poor ecological conditions, followed by scrapers. For example, shredders’ dominance at the J1A (74%) and AL2 (69%) sites was highlighted, as was scrapers’ dominance at the ALB1 (67%) and ALB2 (80%) sites (Figure 4B—centre). The density of macroinvertebrates with parasitic and predatory habits, although not high, was relatively higher in poor ecological conditions than in the other types of ecological conditions, especially parasites, which reached 40% density in site H6 (40 of 69 individuals among all locations sampled). Predators were most abundant in sites ALB1, AL1 and H5 (in increasing order of ecological conditions) with 11%, 15% and 17% relative abundance per site, respectively (Figure 4A,B). PERMANOVA analysis showed that there were significant differences between sampling sites grouped according to the BMWP-Col index with respect to macroinvertebrate functional feeding groups (F = 2.8834; *p* < 0.05). However, pairwise comparisons (post hoc PERMANOVA) revealed that BMWP-Col classes are not significantly different from each other (FDR-adjusted *p* < 0.05) for macroinvertebrate functional feeding groups, most likely due to a lack of statistical power as the groups have small sizes. Meanwhile, in the case of the ABI and AAMBI indices, no significant differences were found between ecological condition classes (Table 3).

At the very bad ecological condition site (AL3), there was only one occurrence of the top-scoring taxa (more sensitive) in the BMWP-Col index (scrapers) (Table 2); however, they accounted for half of the FFG richness found at this site (Figure 3C). There were only two families in the location with a very bad ecological condition. At site AL3, only scrapers and collector–gatherers were found, with a dominance of the latter (71%), and it was the site with the lowest absolute abundance of individuals (14) of all the sites sampled (Figure 4A,B). This result was most possibly related to the existence of a concrete channel at this sampling site.

In summary, in the upper part of the ARB, the FFGs are distributed according to presence/absence, abundance and richness as follows: (i) predator and collector–gatherer taxa are more tolerant to pollution and other environmental stressors according to the BMWP-Col index, and their distribution, although heterogeneous, was higher at sites with poor ecological conditions; (ii) scraper and shredder taxa are more sensitive to disturbances; (iii) macroinvertebrate abundance and richness (and thus FFGs) increase with better ecological conditions; (iv) shredders dominate at sites with poorer ecological conditions, whereas scrapers dominate where ecological conditions are better; and (v) collector–filterers only appear when ecological conditions improve.

## 4. Discussion

The integration of physico-chemical and hydro-morphological variables with information provided by biotic indices (presence/absence or abundance/richness) and ecological functionality (via FFGs) usually allows for a more holistic picture of what is happening in a stream. Multiple site-specific (e.g., flow velocity, width, elevation, depth) and physico-chemical (e.g., nutrients, oxygen, conductivity) variables have been identified as potential parameters that explain biotic processes such as the structure and abundance of macroinvertebrate FFGs. Tropical high-altitude streams are shorter than lowland streams, and precipitation is usually lower; therefore, these are smaller in terms of area or discharge [2]. These small channels can be observed in our study sites (see Figure A4), where, in general, high flow velocities were not observed. From the site-specific variables analysed, the results suggest that a higher elevation is slightly related to sites with better ecological conditions; a similar situation occurs with higher flow velocity (Figure 2). It has been documented that elevation is related to ecological conditions [24,28,38]. Stream velocity seems to be at adequate values according to the climatic and morphological characteristics of this area [21,28,66]. The deep soils rich in organic matter, characteristic of the Andean *páramos*, give rise to narrow streams with low turbulence that flow over beds of fine substrate [2], allowing suitable adaptation of aquatic invertebrates [67,68]. Although depth appeared to be unrelated to ecological condition, it was inversely related to flow velocity. The greater the depth, the lower the turbulence due to lower flow velocity and, therefore, the lower the oxygen concentration (Figure 2). Moreover, channel width seemed to disfavour ecological conditions, as indicated by the BMWP-Col index, and did not match the typical morphology of tropical high-altitude streams [69,70].

Ecuadorian tropical high-altitude ecosystems exhibit broad-scale regional and altitudinal differences in water chemistry. Nitrogen and phosphorus are frequently linked to organic pollutants and nutrient runoff originating from agricultural practices, thereby serving as reliable indicators of the proximity of organic and fertilizer contaminants. Nutrients decrease from higher elevations to lower elevations, the latter with more typically leached and nutrient-poor soils [2]. Studies on 45 streams in the Ecuadorian Andes have indicated that geographic region explained the variation in water chemistry between sites more than altitude or human disturbance of watersheds [71]. Although, on the other hand, the limited data that exist in these high-altitude streams would suggest that nutrient levels in upland areas may not necessarily be different from lowland streams and may be elevated because of anthropogenic activities or nutrient-rich upland soils [2]. In the present work, high nutrient concentrations and conductivity were found to be related to lower ecological conditions (Figure 2). Since the Antisana area is a protected National Park in Ecuador, concentrations of nutrients and other particles (e.g., organic matter or dissolved salts) are not related to human activity [51]. Geologically complex uplands with a high heterogeneity of soil types and recent volcanic activity may show high variability in water chemistry over short distances [2]. Furthermore, mean pH and conductivity values increase from high-altitude streams towards lowlands areas, which would also explain the dissolved salt concentrations in streams in our study area.

Oxygen concentrations at the study sites appeared to be in reasonable ranges according to the type of aquatic ecosystem (DO concentration from 6.9 to 8.8 mg/L and DO saturation from 96.2 to 104.9%). According to the NMDS analysis (Figure 2), it was observed that oxygen was inversely related to the concentration of dissolved nutrients and salts. DO saturation was related to altitude and to a lesser extent to flow velocity. Jacobsen [2] indicated that the high solubility of oxygen in cold waters, such as high-altitude streams, is counteracted by the decrease in atmospheric oxygen pressure at these altitudes. Thus, the oxygen concentration is often affected by the atmospheric partial pressure of oxygen, with the percentage of saturation being a more important variable for stream biota [72]. Although macroinvertebrates did not seem to need special respiratory adaptations for cold, turbulent, oxygen-saturated mountain stream waters [73], more recent findings indicate that at high altitudes, oxygen may be a limiting factor for macroinvertebrates [74]. Since macroinvertebrates obtain oxygen primarily by passive diffusion at their body surface, partial diffusion of oxygen within the animal and the surrounding water is key [75]. In our results oxygen saturation was slightly directly related to altitude (Figure 2), Jacobsen et al. [74] suggest that at 4000 m.a.s.l., the potential oxygen supply for macroinvertebrates would be only one-fifth of that at sea level. Although the oxygen demand of macroinvertebrates is half that at lower altitudes [76], the gap between oxygen supply and demand can be significant, so that in high-altitude streams, macroinvertebrates live near or under oxygen-deficient conditions [2]. One of the adaptations to these high-altitude, oxygen-poor environments may be to increase ventilation, with a preference for microhabitats with faster water currents [74]. In the present study, we found the importance of flow velocity with respect to ecological conditions, with the BMWP-Col score being higher at higher flow velocities. In addition, the exclusion of species poorly adapted to low oxygen saturation can be expected, generating macroinvertebrate assemblages with lower species richness. These assemblages may also be less resilient or more sensitive to a greater decrease in oxygen pressure, e.g., due to pollution from human beings, than assemblages at lower altitudes [38,42,67,74,77,78,79].

In the Antisana River Basin, the best ecological condition (moderate) is found at the sites with a high abundance and richness of macroinvertebrates. This situation was observed at Humboldt and Antisana streams sites (north ARB), each containing more than 100 individuals (except H5 site) belonging to 8 and 13 different taxa, respectively. Most of the study sites would be expected to have reached high values in the BMWP-Col index because they are in a protected, pristine area with almost no pollution. The sites with the best ecological condition were those located in the northern streams, with a medium-high slope on their banks, mixed vegetation (a mixture of grasslands and *páramo* vegetation) and the presence of waterfalls and rapids. Some possible characteristics that explain this circumstance may be that, among the study sites, they are those with better conservation and less disturbance (See Figure A4). Also, the morphology of these streams (higher flow velocity, narrower channel width) and the physico-chemical variables (higher oxygen saturation and lower concentration of salts and nutrients) allow a better environment for macroinvertebrate species [2,20,28,34].

On the other hand, the index values were low at Moyas 3 (AL3) and La Alambrada (ALB1 and ALB2) sites, all of them located in south ARB (Figure 1), with natural and anthropogenic differences with respect to the better-ecological condition sites. At site AL3, only 14 individuals of two taxa were collected, scoring 9 on the BMWP-Col index (very bad class) because it is the only site sampled over a concrete channel. Macroinvertebrates encountered significant challenges in adhering to artificial substrates, resulting in a notable decrease in their abundance and diversity. There were other sampling sites where concrete canal constructions, plastic water tunnels or other infrastructures were observed (33% of the sampled sites). These types of human structures significantly alter aquatic microhabitats; reduce biological dispersal, aquatic community relationships and ecological functionality; and especially diminish the ecological integrity of streams [80,81]. The relationship between the presence of these constructions and the reduction in the diversity of aquatic invertebrates, and therefore worse ecological conditions, is evident [82,83]. Thus, also around the sites ALB1, ALB2, AL1 and AL2, where these infrastructures exist, the ecological condition of the water was poor, and could certainly be better if such infrastructure did not exist. These sites with poor ecological conditions also had steep slopes with eroded areas, probably related to the works for the installation of such constructions. It is well documented that human disturbances (including infrastructure construction) deteriorate the distribution and abundance of different biological groups (including macroinvertebrates), causing habitat fragmentation and loss of the substrate necessary for the proper development of these taxa [81,84]. Erosion, whether due to land use (anthropogenic), steep slopes (natural) or both, also causes problems for aquatic invertebrate groups, as it produces rocky material or fragments of soil that increase the turbidity of the water and worsen the ecological condition of the habitat [83,85].

**Figure A3:**
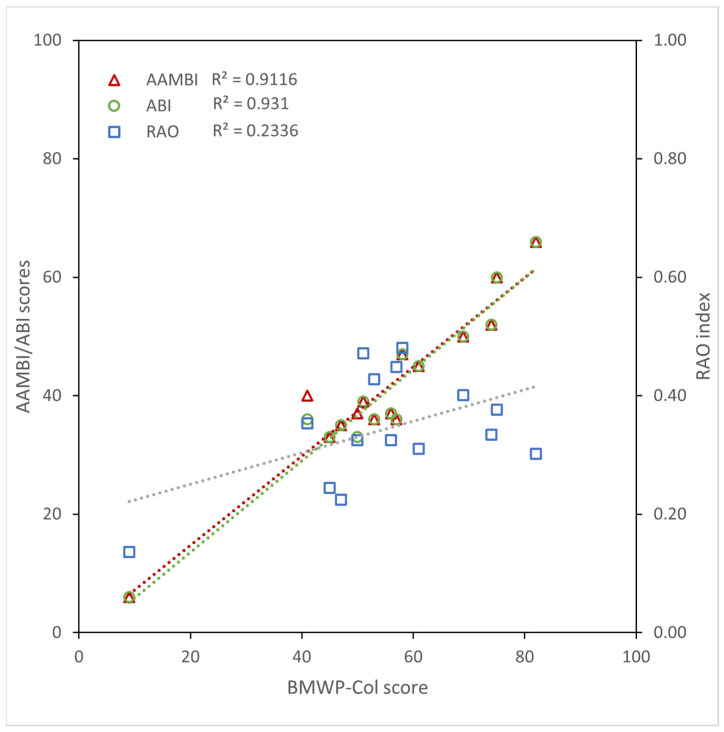
Correlation between the biological indices Biological Monitoring Working Party (BMWP-Col) with Andean Biotic Index (ABI), Andean–Amazon Biotic Index (AAMBI) and Rao’s quadratic entropy index, applied according to the composition of macroinvertebrates and functional feeding traits in the study sites within the Antisana river basin. The degree of linear correlation is indicated together with the R^2^.

Trait-based metrics have been employed in a quarter of freshwater investigations conducted in tropical nations. Among these studies, the primary focus was on the examination of food habits, accounting for a substantial majority of 85% [15]. Even though in high-altitude tropical streams, macroinvertebrates are easily adaptable in terms of feeding habits, the classification of FFGs may be difficult [34,41]. The support of a growing number of studies in the region allows us to overcome this challenge with increasing certainty. The assignment of feeding traits used in the present study was based on fuzzy coding, using several previous studies in the area and attributing to invertebrates the FFG that is most similar and common in these studies [42,43,44,45,46,47,48,49]. Furthermore, in the allocation of feeding traits, it is customary to employ gender-resolution identification. However, studies such as Sotomayor et al. [86] unequivocally demonstrated that there is negligible disparity in the outcomes when employing either family or genus levels. In essence, the family level proves to be satisfactory. Moreover, several of the referenced research papers in the present study have also used the family level as the highest resolution for the application of trait allocation and ecological indices [56,57,58]. Our results show that the sites with better ecological conditions were related to the abundance of scrapers, collector–filterers and collector–gatherers (Figure 2). Scraper, shredder and collector–gatherer species had a high sensitivity score on the BMWP-Col biological index, while collector–gatherers and predators had a low sensitivity score on this index. The association of FFGs with ecological conditions is evident considering that the dominance and diversity of some food traits changes completely when the degree of disturbance increases [15]. If we look at the distribution of the presence/absence macroinvertebrates species (and subsequently FFG) according to the ecological condition of sites, the presence of enhanced ecological conditions was discernible through the increased diversity of traits, notably characterized by a prominent prevalence of scrapers and shredders (Table 2). The sites with the best BMWP-Col scores, namely H2 (82), H1 (75), J1B (74), H3 (69) and H4 (61), had a high taxa richness (12, 12, 13, 11 and 10, respectively). Considering that the distance between the study sites is not long and that most of the streams are lower-order, it would be expected that the composition of the FFGs would be similar. But, as mentioned before, both taxa diversity and abundance varied between study sites. Some variables explaining the difference between good and very poor ecological condition sites were, for example, site-specific factors (altitude, flow velocity), physico-chemical variables (nutrients and salts) and some disturbances (i.e., infrastructure, erosion). To this list must be added the presence of human activity in some study sites, such as rural houses, farms, rural roads (vehicles), tourism and the presence of domestic animals (horses). The sites with such anthropogenic disturbances are H5 (ecological condition of 57) and H6 (51), which despite belonging to the Humboldt stream, where the rest of the sites had better ecological conditions, was in a poor class, with the lowest taxa richness and abundance and, therefore, the lowest FFG diversity. Similarly, among the poor-ecological-condition sites, those that had the highest abundance and diversity of taxa and FFG were sites J1A and J1C because they did not have the disturbances that the rest of the sites had (i.e., infrastructure, horses, estate, etc.). However, the other J1 sites (J1A and J1C) might have better ecological conditions and resemble the very close site J1B, where environmental conditions were better (Figure A3) and also where, despite having lower abundance, taxa richness was higher, and therefore, this site reached a higher BMWP-Col value. Feeding traits had also been modified by these disturbances, with a clear dominance of shredders and collector–gatherers at poor-ecological-condition sites J1A and J1C with no apparent disturbances, while at the remaining poor-ecological-condition sites, there was a more heterogeneous distribution.

Comparing the richness of macroinvertebrate taxa at lower-altitude sites in tropical Ecuador showed that there was a decrease in richness with increasing altitude, even though this was not as clear of a phenomenon as in other taxonomic groups (e.g., fish) [74,87,88]. Previous studies showed that the prevalence of shredders is greater in lower temperatures (e.g., high mountains) [85] and small-size streams [89] and that they change seasonally with the input of allochthonous material from heavy rainfall [90]. The abundance of shredders may also be attributed to the presence of riparian vegetation (in this case, grassland) that allows the presence of shredded leaves [78]. Furthermore, the availability of leaf litter tends to be year-round in tropical areas; however, at lower elevations, a shortage of shredders has been observed and there is not always a clear correlation with the amount of leaf material due to the unpredictable behaviour of shredders, especially in tropical regions [15,91,92]. On the contrary, the decline in shredder populations in sites with improved ecological conditions can be attributed to the presence of a higher streamflow, which has the ability to remove accumulated coarse particulate organic matter (CPOM) stocks. This phenomenon is particularly detrimental to the most vulnerable taxa, leading to alterations in community composition and a decrease in species richness. Consequently, a recolonization process is necessary during the limited dry period in these areas [93]. The abundance of shredders can also be explained by the River Continuum Concept (RCC), which predicts that this functional feeding group (FFG) is most prevalent in headwaters (stream orders 1 to 3). In these areas, there is a low photosynthetic ratio and a significant input of allochthonous CPOM [94]. Scrapers prefer sites with a stable substrate—without sediment release (for grazing) [95], without recent flooding, sites with autotrophic characteristics (non-filamentous algae attached to rocks or other substrates) [33,96,97], high-quality riparian vegetation [91], no high salinity (low conductivity) [37]—and according to the RCC, they are usually located in the midreaches (stream orders 4–6) [94]. Our study sites do not fit with most of these criteria, mainly since most taxa in the reviewed studies show affinity to more than one FFG, with scrapers being one of the most frequent [34]. In addition, the findings are also hindered by three circumstances: lack of autoecology on Ecuadorian high-altitude stream taxa and the degree of taxonomic resolution to determine feeding habits; the particular (environmental and habitat) characteristics of the *páramos*; and the high adaptability and ubiquity of this FFG [15,98,99]. Meanwhile, the environmental characteristics found in the present study, which coincide with the best adaptation of scrapers in the literature, are that these sites have a stable substrate, good riparian quality, low conductivity and are favoured by an environment with little or no human disturbance [46]. The ecological role of scrapers is key in the transfer of matter and energy through the food chain, being the main regulators of primary producers of biomass in these streams [45,100]. In this study, predators are the scarcest FFG (no presence in very poor ecological conditions). The potential correlation between the distribution of predators and low levels of disturbance, such as deforestation, nutrient inputs, intensive land use and organic effluents, in high-altitude environments is a subject of academic interest [15,101,102].

**Figure A4:**
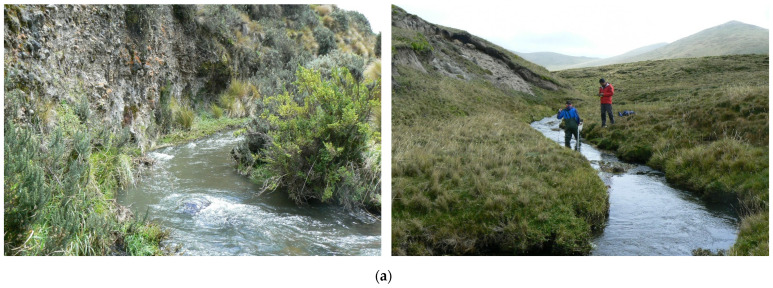

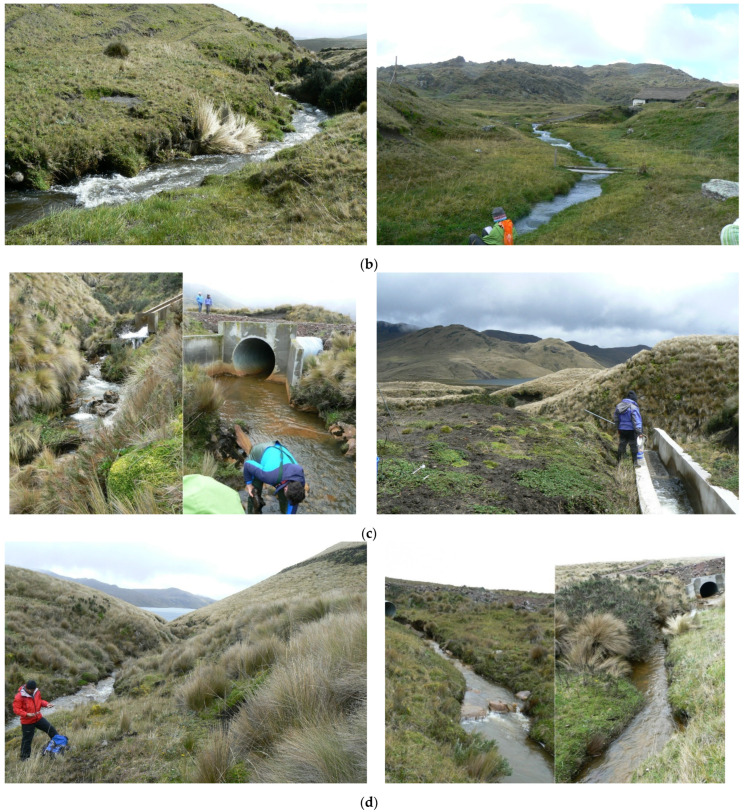
Photographs of the sampling sites on the Antisana River Basin. The letters showed the place where the images were captured: (**a**) Antisana stream, (**b**) Humboldt stream, (**c**) Moyas stream and (**d**) Alambrada stream.

There is a wide range of variables for assessing stream ecological conditions, most of which are physico-chemical and biological. They can often be grouped together to generate indices that reduce the multivariate nature and homogenise units [15,25,27,29]. Beyond the advantages they offer separately (e.g., easy comprehension, qualitative and quantitative characterisation), some authors indicate that it is more accurate to use both of them either in combination or in an integrated manner [42,103,104]. For example, in the present study, although the physico-chemical variables do not indicate water contamination in sites such as AL3, the biological index shows very poor ecological conditions. That is, the composition and abundance of macroinvertebrates might indicate problems in that stream, but sometimes, it might be due to its natural characteristics or the lack of adaptability of taxa to these water systems [18,33], but not to contaminated water as such. In this sense, it is important to indicate that some of the streams sampled in the present study drain naturally or artificially (human infrastructure) into La Mica Lake (Figure 1), and the water is used as drinking water for the city of Quito, so it complies with the quality standards imposed by Ecuadorian regulations [51]. The validity and usefulness of the BMWP-Col index is amply exemplified in some studies, not only in tropical high-altitude streams [38,42,77,91,103,105], but also in other types of aquatic ecosystems [106,107,108,109]. In the present work, for example, site AL3 had a poor ecological condition, as only 14 individuals belonging to two taxa were found, which is explained by the fact that this site is a concrete channel, generating a negative impact on the population of aquatic insects. Therefore, the index supports the management of these streams, indicating that efforts should be made to omit the implementation of these human infrastructures. In the evaluation of rivers and streams, sensitive metrics were utilized in approximately 40% of the research conducted. Among these studies, a majority of 31% employed a variation of the Biological Monitoring Working Party (BMWP) and Average Score Per Taxon (ASPT) methods, as evidenced by sources [15,110]. The application of the BMWP-Col index revealed that a higher diversity of macroinvertebrates corresponded to a more favourable index and, consequently, a better ecological state of the river/stream. However, a weakness that could influence the accuracy, versatility and suitability of this index is that it does not consider the number of individuals but only the presence/absence of macroinvertebrates. Thus, for example, BMWP-Col scores for Amazonian river sites [38] are, in general, higher than those obtained for Antisana sites. However, this does not mean that the Amazonian river sites had better ecological conditions or water quality, as they were exposed to more environmental stressors (lower altitude, land use and human activities), but that they had a higher diversity of taxa (8 to 10 taxa on average per site). Although the richness in the Antisana streams was lower (less variation in macroinvertebrates found) than in Amazonia, the average abundance of individuals per site was higher (111 vs. 95 individuals). The BMWP-Col is not the only index used for tropical Andean freshwater bodies; yet, it is the most widely accepted and applied [15,25,30,42,110,111,112]. Other indices proposed for the area are the Andean Biotic Index (ABI) [25] and the Andean–Amazon Biotic Index (AAMBI) [26], which have similar calculation methods (presence/absence) or are based on the BMWP-Col. Therefore, when comparing them, findings were quite similar (Figure A4), although their classification system slightly varied (Table A2) in our study sites. The variability is mainly due to the number of taxa that are used in the composition of each index and the degree of sensitivity/tolerance assigned to them. In general, the ABI and AAMBI use fewer macroinvertebrate taxonomic groups, although the former is slightly more similar to BMWP-Col. On the other hand, when comparing the BMWP-Col scores with the Rao quadratic entropy, a lower correlation was observed than with ABI and AAMBI, this may be because the calculation of the Rao index [65] differed considerably from how the other three indices were obtained. Rao is based on FFGs, while the others are based on taxa. Therefore, when categorising the sampling sites in this study according to their ecological condition, although all the indices coincide in the very poor class sites, the Rao index seems to have some inconsistencies, with high values in poor ecological condition sites and lower scores in better quality sites (Table A2). The BMWP index is widely utilized and has been globally modified to ensure its relevance to specific regions of interest. The existence of diverse taxa and their varying responses to stressors precludes the establishment of a universal index applicable worldwide. This is evident even within the European Union (EU), which mandates the reporting of the ecological quality of streams and rivers through its Water Framework Directive; yet, EU member states use distinct ecological indices [14,16,109,113]. Despite this, global efforts have been made to unify and generate a common metric not only for tropical areas but globally given the many similarities in the applied methods and efficiency obtained when working with macroinvertebrates as bioindicators worldwide [15]. In this regard, trait-based methodologies hold promise for ecological assessment and exhibit potential for cross-country application, as taxonomic traits may exhibit similar responses to stressors [86,114,115,116,117]. Nevertheless, it is imperative to conduct additional research to ascertain whether taxonomic traits respond uniformly to stressors across different regions. In the context of this research, we posit that a significant stride towards enhancing our comprehension of the diversity and ecological functionality of macroinvertebrates in tropical Andean streams lies in the comparison of biological indices and the evaluation of the precision of presence/absence-based indices. Although probably to guarantee the reliability and advantages that biological indices such as BMWP-Col have, and since they are cost-effective and appropriate tools for biomonitoring, a new Ecuadorian/Latin American index, which takes into account both abundance and richness, is needed. Given the vulnerability and escalating human-induced stressors faced by these high-altitude streams, it is imperative to establish a consistent and comprehensive biomonitoring approach. Our research endeavours contribute to the development of technical and methodological frameworks for investigating various variables, including physical, chemical, hydro-morphological and even biological aspects (such as macroinvertebrates and functional feeding groups). The data generated from the present study can serve as a benchmark for future sampling initiatives in the area, enabling the assessment of long-term impacts resulting from environmental stressors (e.g., hydraulic infrastructures, tourist activities and even climate change).

## 5. Conclusions

The macroinvertebrate community composition within the Antisana River basin in the Ecuadorian Amazon Region exhibited slight variations across different study sites. However, it is worth noting that sites with similar ecological conditions, as determined by the BMWP-Col index, displayed similarities in both taxa abundance and feeding group composition, with only a few exceptions. Considering the challenging environmental conditions of the high-altitude streams, the overall diversity of taxa and functional feeding groups (FFG) tended to be low. Nevertheless, sites with better ecological conditions tended to exhibit higher levels of diversity in both taxa and FFGs. Shredders were generally the most dominant, particularly in areas with elevated nutrient concentrations and conductivity. On the other hand, scrapers represented the second most abundant group, primarily dominating in sites with moderate ecological conditions (upstream and higher oxygen saturation). Collector–gathers were shown to be less sensitive to contamination than the previous two groups but were abundant at sites with better ecological conditions. Predators were almost absent, with small presences in poor-ecological-condition sites. Collector–gatherers, parasites and predators showed more tolerance to the presence of human disturbances (e.g., hydraulic constructions, land use or slope erosion). The present study indicates that the dissolved oxygen saturation, phosphorus and ammonia concentration are significant factors in elucidating the distribution and abundance of taxa and traits. In general, sites exhibiting superior ecological conditions and devoid of external stressors tended to harbour a greater diversity of taxa and functional feeding groups (FFG). The analysis of physico-chemical and, in particular, biological variables revealed that the poorest ecological condition was linked to human hydraulic infrastructure, which significantly restricted the diversity of macroinvertebrates. Although in some sampling sites the ecological condition class was not high, probably showing that the environment is less suitable for macroinvertebrates, this does not mean that the physico-chemical quality was bad (i.e., for drinking water supply). Therefore, utilizing the aforementioned circumstance as an illustrative example, it is plausible to employ this research as a fundamental benchmark and informational resource for potential future bioassessment initiatives. Additionally, it can aid in distinguishing the impact of human-induced disturbances from natural fluctuations, thereby enabling the adjustment of biotic index values for various sections of rivers or the creation of novel ones specifically tailored to the Ecuadorian Andean region. 

## Figures and Tables

**Table 1:** Mean, minimum, maximum and standard deviation of continuous variables measured in 15 sampling sites.

Variable	Mean ± SD	Minimum	Maximum
Dissolved oxygen (mg O_2_·L^−1^)	7.6 ± 0.55	6.9	8.8
Dissolved oxygen saturation (%)	103 ± 2.2	96	105
pH	7.5 ± 0.64	6.1	8.8
Conductivity (µS·cm^−1^)	121 ± 55.6	34	217
${NH}_{3}$ (mg·L^−1^)	0.33 ± 0.214	0.07	0.76
COD (mg·L^−1^)	12 ± 4.1	10	23
${NO}_{3}^{-}$ (mg·L^−1^)	0.81 ± 0.853	0.05	2.80
${NO}_{2}^{-}$ (mg·L^−1^)	0.02 ± 0.023	0.01	0.10
Total nitrogen (mg·L^−1^)	1.04 ± 0.825	0.22	3.60
${PO}_{4}^{3-}$ (mg·L^−1^)	0.29 ± 0.258	0.05	0.93
Total phosphorus (mg·L^−1^)	0.13 ± 0.104	0.05	0.40
Elevation (m.a.s.l.)	4009 ± 31.1	3932	4037
Velocity (m·s^−1^)	0.7 ± 0.31	0.2	1.4
Width (m)	1.63 ± 0.918	0.75	4.00
Depth (m)	0.34 ± 0.097	0.15	0.5

**Table 2:** Macroinvertebrate and functional feeding group composition ordered according to the sensitivity score in function of the increased BMWP-Col ecological conditions of the sampling sites in the Antisana River basin. The horizontal line divides low-sensitivity-score taxa (=tolerance taxa) from the high-sensitivity-score taxa. The bold vertical lines separate the different classes of ecological conditions. p means presence. PA = parasites; CF = collector–filterers; SH = shredders; SC = scrapers; PR = predators; and CG = collector–gatherers. The middle dash indicates that taxa are not assigned a sensitivity value for the BMWP-Col index.

	Site		Al3	A1	Al1	Al2	Alb1	Alb2	H5	H6	J1A	J1C	H1	H2	H3	H4	J1B
Taxa (FFG)		Ecological Condition	Very Bad	Poor									Moderate				
	Sensitivity Score																
Acari (PR)	-					p	p										
Erpobdellidae (PR)	-											p					
Lumbricidae (CG)	-			p					p		p						
Tubificidae (CG)	1												p		p	p	p
Chironomidae (CG)	2		p	p	p	p				p		p	p	p		p	p
Limoniidae (CG)	3						p										
Muscidae (PR)	4				p				p								p
Scirtidae (CG)	4			p		p	p	p		p	p	p	p	p	p		
Ceratopogonidae (PR)	5				p	p	p		p								
Glossiphoniidae (PR)	5					p			p			p			p		p
Dugesiidae (PA)	6			p	p	p		p	p	p	p	p	p	p	p	p	p
Elmidae (SC)	6			p	p	p	p	p	p			p	p	p	p	p	p
Hyallelidae (SH)	7			p	p	p	p	p	p	p	p	p	p	p	p	p	p
Baetidae (SC)	7		p	p	p	p	p	p	p	p	p	p	p	p	p	p	p
Simuliidae (CF)	7				p			p		p	p	p	p	p	p	p	p
Hydroptilidae (SC)	8													p			
Leptoceridae (CG)	8			p				p			p	p	p	p	p	p	p
Limnephilidae (SH)	8			p		p			p	p	p	p	p	p	p	p	p
Hydrobioscidae (PR)	9				p		p		p				p	p		p	p
Gripopterygidae (SC)	10			p						p			p	p	p		

**Table 3:** PERMANOVA (permutational ANOVA) result for macroinvertebrates’ functional feeding groups and environmental variables between sites according to ecological condition indices classes. SS = sum of square; MS = mean square; *df* = degree of freedom. * *p* < 0.05.

Functional Feeding Groups							
	Source	*df*	SS	MS	*F*	N. Perm	*p*
BMWP-Col index							
	BMWP-Col classes	2	0.40049	0.200245	2.8834	9999	0.0148 *
	Residuals	12	0.83337	0.0694475			
	Total	14	1.23387				
ABI index							
	ABI classes	2	0.24140	0.1207	1.4594	9999	0.1626
	Residuals	12	0.99246	0.082705			
	Total	14	1.23387				
AAMBI index							
	AAMBI classes	2	0.18213	0.091065	1.039	9999	0.4163
	Residuals	12	1.05174	0.087645			
	Total	14	1.23387				
Environmental Variables							
BMWP-Col index							
	BMWP-Col classes	2	0.0029954	0.0014977	2.5571	9999	0.0563
	Residuals	12	0.0070285	0.0005857083			
	Total	14	0.0100239				
ABI index							
	ABI classes	2	0.0025451	0.00127255	2.0418	9999	0.1259
	Residuals	12	0.0074788	0.0006232333			
	Total	14	0.0100239				
AAMBI index							
	AAMBI classes	2	0.0020292	0.0010146	1.5229	9999	0.2172
	Residuals	12	0.0079946	0.0006662167			
	Total	14	0.0100239				

## Data Availability

The data presented in this study are available on request from the corresponding author.

## Appendix A

**Table A1:** Inventory of the taxa encountered in the basins, along with their respective total abundance, the number of sampling sites where they were found and their tolerance scores based on BMWP-Col as determined by Alvarez (2005).

Order	Family	Total Abundance	Number of Sampling Sites	BMWP-Col Tolerance Score
Acari	Acari	4	2	-
Amphipoda	Hyalellidae	494	14	7
Arhynchobdellida	Erpobdellidae	1	1	-
Coleoptera	Elmidae	53	12	7
	Scirtidae	60	10	6
Diptera	Ceratopogonidae	5	4	5
	Chironomidae	34	10	2
	Limoniidae	1	1	-
	Muscidae	3	3	5
	Simuliidae	87	10	6
Ephemeroptera	Baetidae	458	15	5
Haplotaxida	Tubificidae	6	4	1
Hirudinida	Glossiphoniidae	6	5	3
Opisthopora	Lumbricidae	14	3	-
Plecoptera	Gripopterygidae	11	5	10
Trichoptera	Hydrobiosidae	14	7	9
	Hydroptilidae	1	1	8
	Leptoceridae	227	9	8
	Limnephilidae	169	11	8
Tricladida	Dugesiidae	97	13	6

**Table A2:** Comparison between the Biological Monitoring Working Party (BMWP-Col), Andean–Amazon Biotic Index (AAMBI), Andean Biotic Index (ABI) and RAO indices applied according to the composition of macroinvertebrates in the study sites within the Antisana river basin. Their classes and scores are also indicated. The altitude is also represented.

**Sampling Code**	**BMWP-Col**	**ABI**	**AAMBI**	**RAO**	**Index**	**Value**	**Class**
					BMWP-Col	>100	Good
A1	58	47	47	0.48		61–100	Moderate
AL1	53	36	36	0.43		36–60	Poor
AL2	50	33	37	0.32		16–35	Bad
AL3	9	6	6	0.14		0–15	Very bad
ALB1	41	36	40	0.35			
ALB2	45	33	33	0.24	ABI	>96	Very good
H1	75	60	60	0.38		59–96	Good
H2	82	66	66	0.30		35–58	Regular
H3	69	50	50	0.40		<35	Bad
H4	61	45	45	0.31			
H5	57	36	36	0.45	AAMBI	>121	Excellent
H6	51	39	39	0.47		90–120	Very good
J1A	47	35	35	0.22		50–89	Good
J1B	74	52	52	0.33		36–49	Regular
J1C	56	37	37	0.32		<35	Bad
